# Comparison of Physical Therapy Utilization and Motion-Related Re-operations Between Isolated Anterior Cruciate Ligament and Multi-Ligament Knee Injuries

**DOI:** 10.7759/cureus.40681

**Published:** 2023-06-20

**Authors:** Cale A Jacobs, Austin V Stone, Darren L Johnson, David C Landy, Caitlin E Conley

**Affiliations:** 1 Orthopedic Surgery, Brigham and Women’s Hospital, Boston, USA; 2 Orthopedic Surgery and Sports Medicine, University of Kentucky, Lexington, USA

**Keywords:** lysis of adhesions, manipulation, complication, multi-ligament knee injury, anterior cruciate ligament

## Abstract

The increased prevalence of postoperative arthrofibrosis after multi-ligament knee injuries (MLKI) compared to isolated anterior cruciate ligament (ACL) injuries has been proposed to be due, in part, to patient factors limiting physical therapy utilization. The purpose of this study was to compare demographic factors, pre- and postoperative physical therapy utilization, and the need for motion-restoring surgery between MLKI and ACL-injured patients. Using the PearlDiver Mariner 151 database, two cohorts matched by age and sex were identified using current procedural terminology (CPT) codes and included those age 16 or greater that underwent isolated ACL (n=3801) vs. MLKI reconstruction (n=3801). The number of pre- and postoperative physical therapy visits was recorded, as was the need for motion-restoring surgery (arthroscopic lysis of adhesions or manipulation under anesthesia). Demographic factors, physical therapy utilization, and the prevalence of motion-restoring surgery were compared between the MLKI and ACL groups using t-tests or chi-square tests, as appropriate. A significantly greater proportion of those with MLKI underwent subsequent motion-restoring surgery (MLKI=412/3081 (13.4%) vs. ACL=84/3081 (2.7%), p<0.001; odds ratio = 5.5 (95% CI: 4.3, 7.0), p<0.0001). Following surgery, less than half of those with MLKI that underwent subsequent motion-restoring surgery attended physical therapy, which was significantly lower than those who did not require motion-restoring surgery (p<0.0001). The prevalence of motion-restoring surgery was significantly greater after MLKI when compared to an isolated ACL injury. While the etiology of arthrofibrosis after MLKI is likely complex, the current results suggest that demographic factors and physical therapy utilization are not solely responsible for the increased risk of arthrofibrosis after MLKI.

## Introduction

Individual patient cohorts have reported that arthrofibrosis is more common following surgical treatment of multi-ligament knee injuries (MLKI) than isolated anterior cruciate ligament (ACL) injuries [1-3]. The increased incidence of postoperative arthrofibrosis after MLKI has been suggested to be due to increased inflammation secondary to greater joint trauma [4, 5]. However, the increased incidence of arthrofibrosis after MLKI may also be influenced by patient factors. Physical therapy utilization is influenced by patient factors including biological sex, socioeconomic status, education, and insurance provider [6,7], and these factors may inherently differ between MLKI and ACL patient populations, thereby resulting in reduced physical therapy utilization and an increased incidence of arthrofibrosis. The purpose of this study was to compare demographic factors, pre- and postoperative physical therapy utilization, and the need for motion-restoring surgery between MLKI and ACL-injured patients. We hypothesized that MLKI patients would have a lower prevalence of commercial insurance and that this would correspond with decreased postoperative physical therapy utilization and an increased prevalence of motion-restoring re-operations.

## Materials and methods

Using the current procedural terminology (CPT) codes, we identified two patient cohorts within the PearlDiver Mariner 151 database. This insurance claims database includes information on more than 151 million orthopedic patients. We identified those having isolated ACL reconstruction, and we operationally defined MLKI in this study as persons undergoing surgical treatment of an ACL tear in addition to another ligamentous procedure on the same day (Table 1) [8]. Patients were included if they were age 16 or older and had continuous insurance coverage for at least six months prior to and 12 months after the index ACL or MLKI procedure. Patients with surgery dates between January 1, 2010, and December 31, 2018, were included. We opted not to include patients with surgeries occurring in either 2019 or 2020 to avoid issues associated with access to orthopedic care and/or physical therapy in response to the COVID-19 pandemic. The ACL and MLKI groups were then matched by age and biological sex.

**Table 1:** Current procedure terminology codes used to identify patients and treatments included in the study.

Description	CPT code(s)
ACL reconstruction	29888
Multiple ligament knee injury	29888 + (27405, 27407, 27409, 27427, 27428, 27429, or 29889)
Motion-restoring surgery (MUA or lysis of adhesions)	27570 or 29884
Physical therapy	97001, 97005, 97006, 97010, 97014, 97016, 97022, 97032, 97035, 97110, 97112, 97113, 97116, 97124, 97140, 97150, 97161, 97162, 97163, 97164, 97530, or 97750

The outcome variables of interest were physical therapy utilization and the need for motion-restoring surgery. Similar to Burroughs et al. [9], the number of pre- and postoperative physical therapy visits was identified using CPT codes (Table 1). For the purposes of this study, we assessed physical therapy utilization in the three months prior to and six months following surgery [10]. Motion-restoring surgery was defined as either arthroscopic lysis of adhesions (CPT 29884) or manipulation under anesthesia (CPT 27570). Age, biological sex, and insurance provider were also extracted for each patient.

Demographic factors, the number of pre- and postoperative physical therapy visits, and the prevalence of motion-restoring surgery were compared between the age- and sex-matched groups of ACL and MLKIs using chi-square or t-tests as appropriate. Within the MLKI cohort, subgroup analyses were also performed to compare patients that underwent a subsequent motion-restoring surgery to those that did not. It should be noted that large sample sizes create the opportunity for small group differences to be statistically significant but not clinically meaningful. We included p-values to provide context, but one should be cognizant of the relationship between sample size and statistical significance when interpreting the results.

## Results

Age- and sex-matched groups of 3081 MLKI and 3081 isolated ACL-reconstructed patients met the study inclusion criteria (Table 2). Significantly fewer patients in the MLKI group had commercial insurance when compared to those with isolated ACL injuries (84.6% vs. 89.5%, p<0.001). A greater proportion of patients with MLKI utilized physical therapy both prior to and following surgery (Table 2). For those that utilized physical therapy, the MLKI group averaged one additional preoperative visit (p<0.001); however, the number of postoperative visits did not differ between the MLKI and ACL groups (Table 2).

**Table 2:** Comparison of patient factors, physical therapy utilization, and the need for motion-restoring surgery (arthroscopic lysis of adhesions or manipulation under anesthesia) between those with anterior cruciate ligament injury or multi-ligament knee injuries.

Variable	MLKI	ACL	p-value
N	3081	3081	-
Age (y)	32.2 ± 12.8	32.2 ± 12.8	>0.99
Females (%F)	1414 (45.9% F)	1414 (45.9% F)	>0.99
Commercial insurance	2608 (84.6%)	2756 (89.5%)	<0.001
Patients that utilized preoperative PT	675 (21.9%)	586 (19.0%)	0.005
Mean preoperative PT visits/patient	6.0 ± 5.7	7.0 ± 4.5	<0.001
Patients that utilized postoperative PT	1844 (59.9%)	1689 (54.8%)	<0.001
Mean postoperative PT visits/patient	18.5 ± 13.6	19.1 ± 12.6	0.19
Motion-restoring surgery	412 (13.4%)	84 (2.7%)	<0.001

Subsequent motion-restoring surgery was more common after MLKI than ACL reconstruction (MLKI = 412/3081 (13.4%) vs. ACL = 84/3081 (2.7%), p<0.001). This corresponded with a significantly greater odds ratio of undergoing motion-restoring surgery following MLKI (odds ratio = 5.5 (95% CI: 4.3, 7.0), p<0.0001). In the MLKI group, those that underwent motion-restoring surgery were significantly older and more often female, with fewer patients having commercial insurance (Table 3). Prior to surgery, a greater number of those with MLKI who would require subsequent motion-restoring surgery utilized physical therapy and attended a significantly greater number of visits. Following surgery, less than half of the patients who underwent subsequent motion-restoring surgery attended physical therapy, which was significantly lower than the number of patients who did not require motion-restoring surgery. However, the subset that attended physical therapy attended a greater number of physical therapy visits during the first six weeks, three months, and six months after their index MLKI surgery (Table 3).

**Table 3:** Comparison of patients with multi-ligament knee injuries that did vs. did not require motion-restoring surgery (arthroscopic lysis of adhesions or manipulation under anesthesia).

Variable	Reoperation	No reoperation	p-value
N	412	2669	-
Age	37.2 ± 13.9	31.4 ± 12.5	<0.001
Females (%F)	216 (52.4% F)	1198 (44.9% F)	0.004
Commercial insurance	320 (77.7%)	2286 (85.7%)	<0.001
Patients who utilized preoperative PT	130 (31.6%)	613 (23.0%)	<0.001
#Preoperative PT visits/patient	13.2 ± 10.6	6.9 ± 5.7	<0.001
Patients who utilized postop PT (six weeks)	179 (43.4%)	1470 (55.1%)	<0.001
Mean postop PT visits/patient (six weeks)	13.6 ± 10.6	8.2 ± 4.5	<0.001
Patients that utilized postop PT (three months)	193 (46.8%)	1587 (59.5%)	<0.001
Mean postop PT visits/patient (three months)	21.2 ± 17.2	14.3 ± 8.8	<0.001
Patients that utilized postop PT (six months)	202 (49.0%)	1662 (62.3%)	<0.001
Mean postop PT visits/patient (six months)	25.4 ± 23.5	18.3 ± 13.3	<0.001

## Discussion

The purpose of this study was to compare pre- and postoperative physical therapy utilization and the need for motion-restoring surgery between those with either MLKI or isolated ACL injuries. When compared to those with isolated ACL injuries, fewer patients in the MLKI group had commercial insurance. However, while subsequent motion-restoring surgery was significantly more common after MLKI, physical therapy utilization was also greater among the MLKI group both prior to and following surgery compared to those with isolated ACL injuries. Both the number of patients who utilized physical therapy and the mean number of physical therapy visits were greater for those with MLKI vs. ACL injuries. This is not overly surprising due to the increased injury severity and complexity of MLKI surgical procedures as opposed to isolated ACL injuries.

In the current study, 13.4% of patients treated for MLKI underwent subsequent manipulation under anesthesia or arthroscopic lysis of adhesions, compared to 2.7% of the sex- and age-matched group with isolated ACL injuries (p<0.0001, OR = 5.5). Systematic reviews and large single-center series have reported a greater prevalence of 11.2% and 4.5% for MLKI and isolated ACL injuries, respectively [1,5]. However, the current results are on par with another study that utilized an insurance claims database. Werner et al. reported the prevalence of manipulation and/or lysis of adhesions to range from 2.76% to 14.0% for MLKI and between 0.83% and 1.52% for isolated ACL injuries, depending on meniscal status, concomitant injuries, and treatment [11]. Despite the discrepancies between insurance claim databases and clinical results, it does appear that MKLIs result in a greater prevalence of arthrofibrosis requiring subsequent surgical treatment.

The etiology of postoperative arthrofibrosis is likely complex, and the current results indicate patient factors and/or physical therapy utilization do not explain the increased risk of arthrofibrosis after surgical treatment of MLKIs. This suggests that the underlying mechanisms may be biological and/or socioeconomic in nature. The comparison of physical therapy utilization among those who underwent subsequent motion-restoring surgery after MLKI reveals the two patient subsets that we see clinically: those with stiffness secondary to arthrofibrosis and those with postoperative stiffness that may be secondary to underutilization or poor adherence to physical therapy. Both prior to and following the index MLKI procedure, significantly fewer patients who would later undergo motion-restoring surgery attended physical therapy. Additionally, significantly fewer patients in this subset had commercial insurance. This may point toward the development of postoperative stiffness due to underutilization or reduced access to physical therapy due to socioeconomic factors or other social determinants of health [6,7,12,13]. On the contrary, those who required reoperation and did attend physical therapy attended a significantly greater number of visits both prior to and after the index MLKI procedure than the MLKI patients who did not undergo motion-restoring surgery. This may be indicative of the subset of patients with a dysregulated inflammatory response, greater effusion, and/or genetic predisposition to develop postoperative arthrofibrosis [14-19]. This group may not respond as well to conventional physical therapy and then require surgical intervention to restore motion.

That said, the underlying mechanisms remain unknown but are theorized to be potentially related to a greater inflammatory response due to the increased severity of the injury, increased or persistent inflammation after MLKI reconstruction secondary to more involved surgical procedures, as well as prolonged immobilization that may be required to protect the reconstruction in the early postoperative period. Both the postoperative inflammatory response and rehabilitation protocols will likely differ based on the procedures performed and structures involved, and the primary goal of early postoperative rehabilitation must be to restore the range of motion without overstressing the involved tissues [20]. Whether treating a single ligament or MLKI reconstruction, safely restoring the range of motion is imperative to reduce the risk of arthrofibrosis.

Coding errors may have an impact on the results, as with all insurance claims database studies. Individual patient data are also not available, which limits the ability to perform multivariable regressions to assess risk factors for motion-restoring surgery after isolated ACL or MLKI reconstruction. Finally, the lack of a clear definition of arthrofibrosis and the variability in the indications for motion-restoring surgery have been well documented [21]. Future work is necessary to elucidate both the underlying mechanisms and create more consistent treatment algorithms.

## Conclusions

The prevalence of motion-restoring surgery was significantly greater after MLKI when compared to an isolated ACL injury. While the etiology of arthrofibrosis after MLKI is likely complex, the current results suggest that demographic factors and physical therapy utilization are not solely responsible for the increased risk of arthrofibrosis after MLKI. The underlying mechanisms may then be biological and/or physiological in nature, thereby providing potentially modifiable treatment targets. Future work is necessary to elucidate whether the increased risk of arthrofibrosis for those with MLKIs is related to a greater inflammatory response due to the increased severity of the injury, increased or prolonged inflammation after MLKI reconstruction secondary to more involved surgical procedures, and/or prolonged immobilization that may be required to protect the reconstruction in the early postoperative period.
